# Cytoplasmic location of factor-inhibiting hypoxia-inducible factor is associated with an enhanced hypoxic response and a shorter survival in invasive breast cancer

**DOI:** 10.1186/bcr1838

**Published:** 2007-12-20

**Authors:** Ern Yu Tan, Leticia Campo, Cheng Han, Helen Turley, Francesco Pezzella, Kevin C Gatter, Adrian L Harris, Stephen B Fox

**Affiliations:** 1 Nuffield Department of Clinical Laboratory Sciences, John Radcliffe Hospital, Oxford OX3 9DU, UK; 2 Cancer Research UK Molecular Oncology Laboratory, Weatherall Institute of Molecular Medicine, John Radcliffe Hospital, Oxford OX3 9DU, UK; 3 Pathology, Peter MacCallum Cancer Centre, St Andrews Place, East Melbourne 3002, Australia

## Abstract

**Introduction:**

Hypoxia-inducible factor (HIF)-1α levels in invasive breast carcinoma have been shown to be an adverse prognostic indicator. Cellular HIF-1α activity is regulated by factor-inhibiting hypoxia-inducible factor 1 (FIH-1). In hypoxia, FIH-1 hydroxylation of Asn803 within the C-terminal transactivation domain does not occur and HIF-1α forms a fully active transcriptional complex. The present study investigates the role of FIH-1 in invasive breast carcinoma and its correlation with hypoxia.

**Methods:**

Microarrayed tissue cores from 295 invasive carcinomas were stained for FIH-1, for HIF-1α and for carbonic anhydrase 9. FIH-1 expression was correlated with standard clinicopathological parameters and with the expression of the surrogate hypoxic markers HIF-1α and carbonic anhydrase 9.

**Results:**

FIH-1 was positive in 239/295 (81%) tumours, 42/295 (14%) exclusively in the nucleus and 54/295 (18%) exclusively in the cytoplasm. Exclusive nuclear FIH-1 expression was significantly inversely associated with tumour grade (*P *= 0.02) and risk of recurrence (*P *= 0.04), whereas exclusive cytoplasmic FIH-1 was significantly positively associated with tumour grade (*P *= 0.004) and carbonic anhydrase 9 expression (*P *= 0.02). Patients with tumours that excluded FIH-1 from the nucleus had a significantly shorter survival compared with those with exclusive nuclear expression (*P *= 0.02). Cytoplasmic FIH-1 expression was also an independent poor prognostic factor for disease-free survival.

**Conclusion:**

FIH-1 is widely expressed in invasive breast carcinoma. As with other HIF regulators, its association between cellular compartmentalization and the hypoxic response and survival suggests that tumour regulation of FIH-1 is an additional important mechanism for HIF pathway activation.

## Introduction

Regions of hypoxia are common in breast carcinoma [1,2] as the rate of nutrient and oxygen delivery is often insufficient to meet the high metabolic demands of neoplastic cells. The neoplastic cells can adapt to this hostile microenvironment using the activation of hypoxia-induced genes for angiogenesis, glycolysis and other processes advantageous to cell proliferation and survival. The activation of these hypoxia-induced genes centres on the levels of hypoxia-inducible factor (HIF) 1 within the tumour cell [3]. HIF-1 is a heterodimer, consisting of a HIF-1α subunit and a HIF-1β subunit. While HIF-1β is constitutively expressed, HIF-1α levels are tightly regulated with rapid upregulation and degradation [4].

It is therefore not surprising that HIF-1α has been identified in breast tumours and is frequently implicated in altering their behaviour. Tumour cells in perinecrotic regions of ductal carcinoma *in situ *lesions, where HIF-1α levels are high, exhibit a more aggressive phenotype, with loss of differentiation and downregulation of oestrogen-receptor (ER) expression [5,6]. High HIF-1α expression has been demonstrated to be an adverse prognostic indicator, being associated with reduced disease-free survival and overall survival [7,8], and also with an increased risk of metastasis and early recurrence [9].

HIF-1α levels are modulated by post-translational hydroxylation that is dependent on cellular oxygen levels. Two mechanisms involving members of the Fe(II) and 2-oxoglutarate-dependent dioxygenases have been described to date. The prolyl hydroxylase domain enzymes (PHD1, PHD2 and PHD3) catalyse the hydroxylation of conserved proline residues P402 and P564 within the oxygen-dependent degradation domain (part of the N-terminal transcriptional activation domain (TAD)) of HIF-1α [10,11]. This facilitates HIF-1α recognition by Von-Hippel-Lindau protein and subsequent degradation by the E_3 _ubiquitin ligase complex [12,13]. In the absence of cellular oxygen and hydroxylation, HIF-1α subunits are not targeted for proteasome degradation and are able to translocate into the nucleus, where they associate with the HIF-1β subunit. Subsequent recruitment of a number of cofactors including p300, with the C-terminal TAD of HIF-1α [14-16], enables formation of the fully active transcriptional complex.

Factor-inhibiting hypoxia-inducible factor 1 (FIH-1) gives a further level of control. FIH-1 catalyses the hydroxylation of a conserved asparagine residue Asn803 within the C-terminal TAD under normoxic conditions [17,18]. FIH-1 interaction at the C-terminal TAD associates with Von-Hippel-Lindau protein bound at the N-terminal TAD to form a ternary complex that blocks p300 interaction, resulting in repression of C-terminal TAD activity [19]. It has been postulated further that PHD hydroxylation of the conserved proline resides within the N-terminal TAD facilitates Von-Hippel-Lindau protein binding that in turn promotes FIH-1 recruitment to the C-terminal TAD, where it hydroxylates the conserved asparagine residue [20].

In normoxia, therefore, PHD and FIH-1 enzymes act synergistically to degrade and inactivate HIF-1α, restricting HIF-1α activity within the cell to a minimum. As cellular oxygen levels decrease, the PHD enzymes have limited oxygen for hydroxylation and no longer hydroxylate the N-terminal TAD, leading to stabilization and accumulation of the HIF-1α subunit [10,11]. Nevertheless, FIH-1 remains active at this stage and continues to repress C-terminal TAD activity until conditions of severe hypoxia occur, where FIH-1 also fails to hydroxylate the asparagine residue in the C-terminal TAD and releases C-terminal TAD repression [21,22]. This graded response to increasingly severe hypoxia suggests that FIH-1 may have a crucial function as one of the final checks on HIF-1α transcriptional activity.

We have previously demonstrated that FIH-1 is strongly expressed in both the luminal epithelial cells and myoepithelial cells in the normal breast [23]. FIH-1 expression was mostly cytoplasmic, with some weak nuclear staining in a proportion of cells. Breast carcinoma cells showed not only strong expression of FIH-1 in the cytoplasm, but in the nucleus as well [23]. FIH-1 expression was also noted in nonepithelial elements, such as stromal fibroblastic cells, vascular smooth muscle cells and infiltrating inflammatory cells.

To better understand the regulation of HIF-1α in breast carcinoma, we have examined the expression of FIH-1 in a large characterized series of breast carcinomas and have correlated this with standard clinicopathological parameters and various markers of hypoxia. In view of the significant upregulation of FIH-1 expression within the nucleus of tumour cells compared with normal breast tissues, we have also analysed the FIH-1 expression in these two subcellular compartments separately. The subcellular localization of FIH-1 was found to be associated with differing prognostic significance. Tumours expressing FIH-1 exclusively within the nucleus were associated with low tumour grade and a reduced risk of recurrence, while tumours expressing FIH-1 only in the cytoplasm were more likely to express carbonic anhydrase 9 (CA9) and were associated with high tumour grade and poor disease-free survival.

## Materials and methods

### Patient and tumour characteristics

Microarrayed tissue 1 mm cores from 295 invasive breast carcinomas were collected from patients who underwent surgery at the John Radcliffe Hospital, Oxford, UK. Four separate cores were obtained from each tumour specimen; necrotic regions were avoided. The present study has Ethical Committee approval (number C02.216). Cores that were incomplete or that did not include tumour epithelial cells were excluded.

Only patients with operable breast carcinoma were included in the study. No participants had received any neoadjuvant therapy. Information of the patient characteristics, including age, tumour size, tumour grade, histology, nodal status, ER status and human epidermal growth factor receptor 2 (HER2) status, were collected from clinical and pathological records. The median age of the patients was 57 years (range, 29–90 years).

Seventy-one per cent of invasive tumours were classified as invasive ductal carcinoma of no specific type, 7% as invasive lobular carcinoma, and 8% as other histological types (data were unavailable for the remaining 14% of tumours). The median tumour grade according to the Bloom and Richardson criteria was 2, and the median tumour size was 20 mm (range, 2–230 mm). Forty-three per cent of tumours were node-positive, 77% were ER-positive and 13% were HER2-positive. Patients younger than 50 years of age with node-positive or ER-negative tumours or with tumours >3 cm in size were given adjuvant chemotherapy (cyclophosphamide, methotrexate and 5-fluorouracil). Patients with hormone-responsive tumours who were older than 50 years of age were given endocrine therapy. Over a median follow-up period of 105 months (range, 5.1–161.2 months), there were 108 relapses and 114 breast cancer-related deaths.

### Immunohistochemistry

Formalin-fixed, paraffin-embedded tissue sections (4 μm) of microarrayed invasive carcinomas were dewaxed in citroclear and were rehydrated in graduated ethanol solutions. Endogenous peroxidase activity was blocked with 0.5% hydrogen peroxide for 30 minutes, and nonspecific binding with 2.5% normal horse serum for 20 minutes. The respective primary antibodies FIH-1 antibody (clone FIH162c, mouse IgG_2k _monoclonal antibody; Nuffield Department of Clinical Laboratory Sciences, Oxford, UK) [23], CA9 antibody (clone M75; gift from J Pastorek, Institute of Virology, Slovak Republic) [24], and HIF-1α antibody (clone ESEE 122, mouse monoclonal IgG_1_; Nuffield Department of Clinical Laboratory Sciences) [25] were then applied at room temperature. Antibody details are presented in Table 1. Substitution of the primary antibody with PBS served as a negative control. Transfected COS-1 cells expressing FIH-1 were used as a positive control. The primary antibody was followed by application of the Envision kit secondary antibody (Dako A/S, Glostrup, Denmark) for 30 minutes, and the peroxidase reaction was developed using diaminobenzidine provided in the kit. The slides were then counterstained in H & E and mounted in aqueous mountant. HER2 staining was assessed with the Hercept test kit (Dako A/S).

**Table 1:** Details of primary antibodies used

Antibody	Details of antibody	Antigen retrieval	Dilution	Incubation period (hours)	Reference
Factor-inhibiting hypoxia-inducible factor 1 (FIH162c)	Mouse monoclonal IgG_2k _(Nuffield Department of Clinical Laboratory Sciences, Oxford, UK)	None	Neat supernatant	1.5	[23]
Hypoxia-inducible factor 1α (ESEE122)	Mouse monoclonal IgG_1 _(Nuffield Department of Clinical Laboratory Sciences)	Pressure cook for 3 min in ethylenediamine tetraacetic acid, pH 8.0	1:100	4	[24]
Carbonic anhydrase 9 (M75)	Mouse monoclonal (from J. Pastorek, Institute of Virology, Slovak Republic)	None	1:50	0.5	[25]

Scoring was performed by two observers simultaneously. The level of staining for FIH-1 was scored with respect to the percentage of cells expressing the protein and the intensity of staining in both the nucleus and cytoplasm. The scoring system for intensity was: 0, no staining; 1, weak staining; 2, moderate staining; and 3, strong staining. The scoring system for the percentage of cells was: 0, no cells staining positive; 1, <10% cells staining positive; 2, 11–50% positive cells; 3, 51–80% positive cells; and 4, >80% positive cells.

Tumours with an intensity score ≥2 and a percentage score ≥1 were considered positive in the statistical analysis. Although some cytoplasmic staining was also observed in tumours stained with HIF-1α, only tumours demonstrating any nuclear staining were considered positive in the analysis. Cytoplasmic HIF-1α staining was assessed in relation to FIH-1 expression; tumours expressing HIF-1α in the cytoplasm with intensity ≥2 were considered positive in this analysis. We have previously shown that tissue microarrays are representative of the whole tissue section for HIF-1α evaluation [26].

There is no general consensus for CA9 regarding a cutoff threshold, and various authors have used different cutoff thresholds [27-29]. Since a cutoff threshold of 10% is commonly used for many clinical markers, such as ER and progesterone receptor status [30,31], we considered tumours demonstrating membranous staining in ≥10% of cells to be positive in the analysis.

### Statistical methods

Correlation between FIH-1 in a particular subcellular compartment, the other hypoxic markers as well as the various clinicopathological parameters were evaluated using either the chi-square test or Fisher's exact test where appropriate. Correlation with tumour grade was evaluated using the chi-square test for trend. Kaplan–Meier survival curves were calculated using tumour recurrence (defined as the first reappearance of a tumour at any site following definitive treatment) as the endpoint. Disease-free survival between groups was compared using a log-rank test. All univariate and survival analyses were performed with GraphPadPrism version 4 (GraphPad Software Inc., San Diego, CA, USA).

In addition, the Cox proportional hazard regression model was used to identify independent prognostic factors for disease-free survival. This was carried out using the Stata Package release 8.1 (Stata Corporation, College Station, TX, USA). A full model was first created to include all potentially important explanatory variables. At each step, the variable with the smallest contribution to the model was removed, until a final backward stepwise model was obtained. A two-tailed test was used in all analyses and *P *< 0.05 was considered statistically significant.

## Results

### Factor-inhibiting hypoxia-inducible factor-1 expression in invasive breast carcinoma

**Figure 1:**
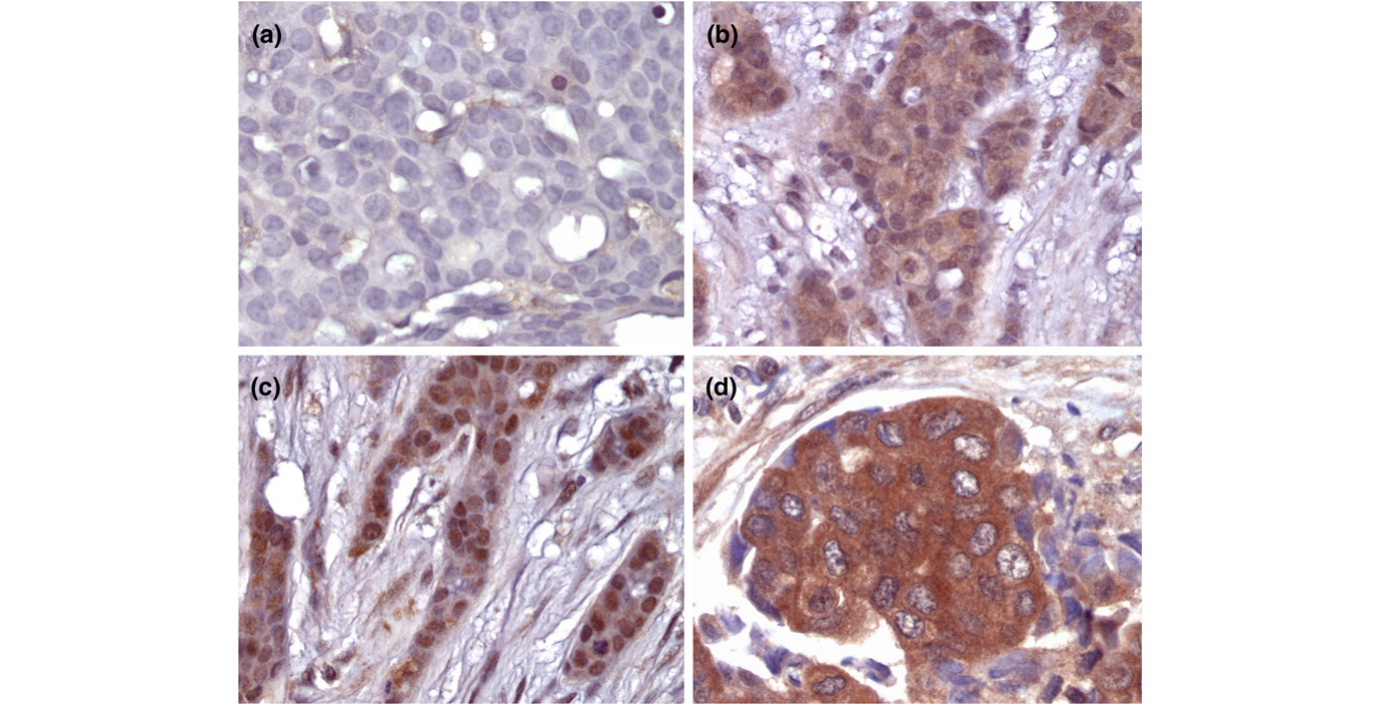
**Immunohistochemistry of factor-inhibiting hypoxia-inducible factor 1 in breast carcinomas**. **(a) **Negative tumour staining. **(b) **Weak tumour cytoplasmic and nuclear staining. **(c) **Strong tumour nuclear staining. **(d) **Strong tumour cytoplasmic staining.

FIH-1 was widely expressed among the invasive breast carcinomas analysed (Figure 1). Eighty-one per cent of tumours (239/295) stained positive for FIH-1, either in the cytoplasm, in the nucleus or in both compartments (Table 2). FIH-1 showed strong expression in the cytoplasm of a large proportion of tumour cells, with a median cytoplasmic intensity of 2 and a median percentage of more than 80% of cells. Nineteen per cent of tumours (56/295) were negative for FIH-1. The majority of the tumours (48.5%) expressed FIH-1 both in the cytoplasm as well as in the nucleus. FIH-1 was expressed exclusively in the cytoplasm in 18% (54/295) of tumours, and exclusively in the nucleus in 14% (42/295) of tumours (Table 2). Nuclear FIH-1 expression had a median nuclear intensity of 2 and a median percentage of 50–80% of cells.

**Table 2:** Subcellular location of factor-inhibiting hypoxia-inducible factor 1 in tumour cells

Subcellular location	Number (%) of cores
Present in cytoplasm only	54 (18%)
Present in nucleus only	42 (14%)
Present in both cytoplasm and nucleus	143 (48%)
Negative for factor-inhibiting hypoxia-inducible factor 1	56 (19%)

Apart from the tumour epithelial cells, FIH-1 was also widely expressed in nonepithelial elements within the tumours. FIH-1 was expressed in the stroma of 78% of tumours, in the vasculature of 81% of tumours and in the infiltrating inflammatory cells in 74% of tumours.

### Correlation of factor-inhibiting hypoxia-inducible factor 1 with clinicopathological parameters and hypoxia markers

Any nuclear FIH-1 expression was inversely associated with tumour grade (*P *= 0.02). Exclusive nuclear FIH-1 expression was also significantly inversely associated with tumour grade (*P *= 0.02). Nuclear FIH-1 expression was not significantly associated with patient age, tumour size, nodal status, ER or HER2 status (*P *> 0.05) (Table 3). Any cytoplasmic or exclusive cytoplasmic FIH-1 expression was significantly positively associated with tumour grade (*P *= 0.05 and *P *= 0.004, respectively). There was no significant correlation between any cytoplasmic or exclusive cytoplasmic FIH-1 expression and patient age, tumour size, nodal status, ER and HER2 status (*P *> 0.05) (Table 3).

**Table 3:** Clinicopathological parameters of the series of 295 invasive breast carcinomas together with correlation analyses of cytoplasmic and nuclear factor-inhibiting hypoxia-inducible factor-1 (FIH-1) expression

	Exclusive nuclear FIH-1 expression (*n *= 42)	Nuclear and cytoplasmic FIH-1 expression (*n *= 143)	Exclusive cytoplasmic FIH-1 expression (*n *= 54)	Nuclear and cytoplasmic FIH-1-negative (*n *= 56)
Median patient age (years)	57	56	58	56
Median tumour size (mm)	18	20	25	20
Tumour grade				
1	12	24	3	8
2	12	43	17	15
3	8	35	21	12
Nodal status				
Negative	23	77	32	35
Positive	19	64	22	20
Oestrogen receptor status				
Negative	8	30	14	20
Positive	34	113	40	35
Human epidermal growth factor receptor 2 status				
Negative	25	95	43	23
Positive	3	17	6	2
Hypoxia-inducible factor 1α				
Negative	10	41	25	22
Positive	32	82	28	20
Carbonic anhydrase 9				
Negative	34	111	33	22
Positive	3	14	11	4
Recurrence				
Negative	32	88	28	37
Positive	9	54	26	18

*n *< 295 data not available.

Any nuclear FIH-1 expression was inversely correlated with CA9 expression (*P *= 0.04), with tumours negative for CA9 also being twice as likely to express FIH-1 within the nucleus (*P *= 0.04, odds ratio (OR) = 2.22, 95% confidence interval (CI) = 0.21–0.96) (Table 3). No significant correlation was observed between exclusive nuclear FIH-1 expression and CA9 (*P *= 0.43). Exclusive cytoplasmic FIH-1 expression, however, was significantly associated with CA9 expression (*P *= 0.02, OR = 2.65, 95% CI = 1.17–6.02) (Table 3).

The relationship between FIH-1 and HIF-1α was also assessed. Nuclear FIH-1 expression showed a significant positive correlation with nuclear HIF-1α expression, being twice as likely to be expressed in HIF-1α-positive tumours (*P *= 0.004, OR = 2.11, 95% CI = 1.26–3.52) (Table 4). There was no significant correlation between nuclear FIH-1 expression and cytoplasmic HIF-1α expression (*P *= 0.68). Cytoplasmic FIH-1 expression was significantly associated with cytoplasmic HIF-1α expression (*P *= 0.03, OR = 2.25, 95% CI = 1.06–4.75) but not with nuclear HIF-1α expression (Table 4).

**Table 4:** Univariate analysis of nuclear and cytoplasmic factor-inhibiting hypoxia-inducible factor 1 (FIH-1) expression with hypoxia-inducible factor 1α (HIF-1α) expression

	Nuclear FIH-1-positive (*n *= 186)	Nuclear FIH-1-negative (*n *= 109)	*P *value	Cytoplasmic FIH-1-positive (*n *= 197)	Cytoplasmic FIH-1-negative (*n *= 98)	*P *value
Nuclear HIF-1α			0.004			0.93
Negative	52	48		66	32	
Positive	114	50		110	52	
Cytoplasmic HIF-1α			0.68			0.03
Negative	129	72		130	68	
Positive	33	21		43	10	

*n *< 295 data not available.

### Correlation of hypoxia-inducible factor 1α with clinicopathological parameters and survival

**Figure 2:**
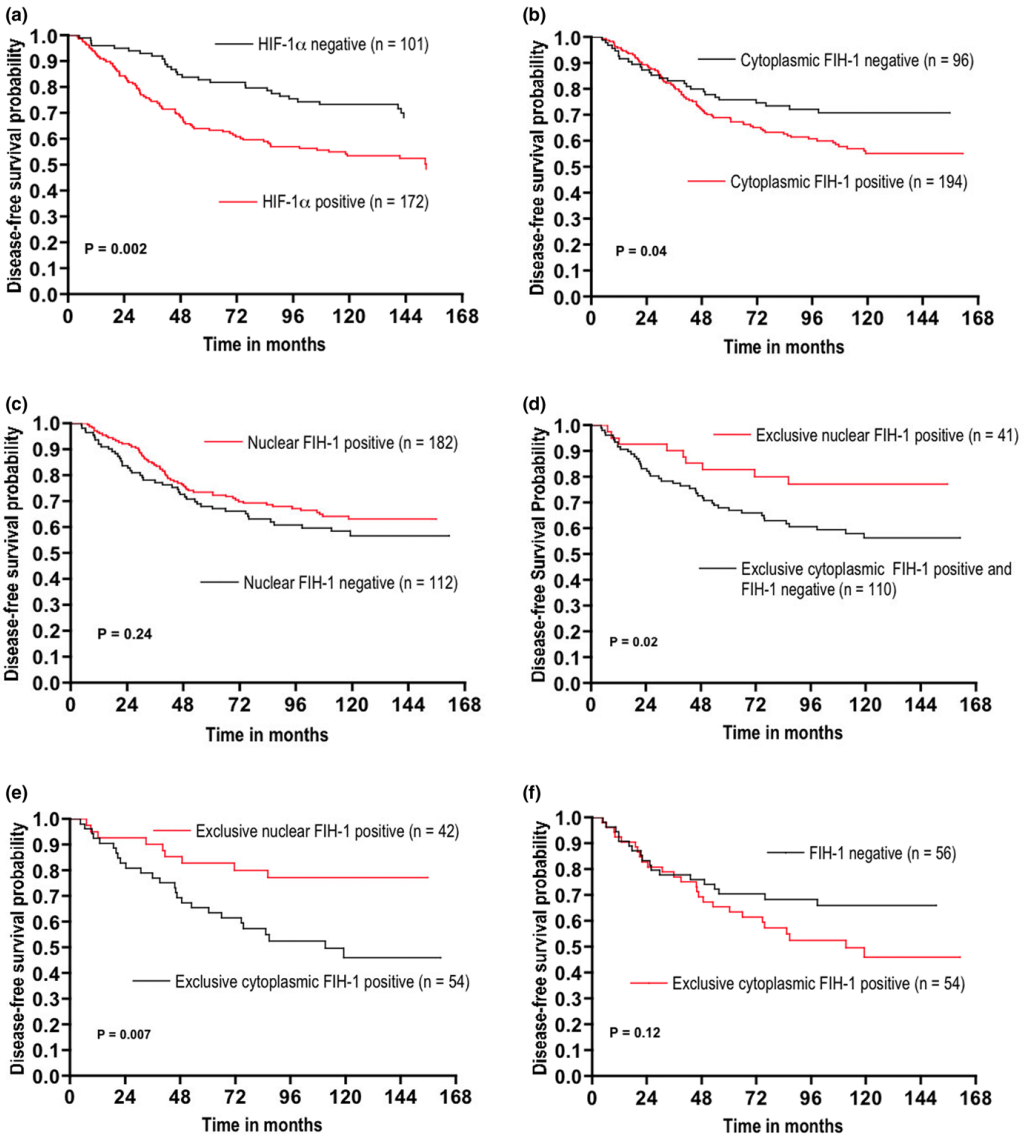
**Kaplan–Meier curves of disease-free survival**. **(a) **Stratifying patients by hypoxia-inducible factor-1α (HIF-1α) expression. **(b) **Stratifying patients by cytoplasmic factor-inhibiting hypoxia-inducible factor-1 (FIH-1) expression. **(c) **Stratifying patients by nuclear FIH-1 expression. **(d) **Stratifying patients by exclusive nuclear FIH-1 expression (functional), and by exclusive cytoplasmic FIH-1 expression and FIH-1 negativity (nonfunctional). **(e) **Stratifying patients by exclusive cytoplasmic FIH-1 expression (nonfunctional), and by exclusive nuclear FIH-1 expression (functional). **(f) **Stratifying patients by exclusive cytoplasmic FIH-1 expression and tumours negative for FIH-1.

Although HIF-1α expression did not correlate with tumour size, tumour grade or ER and HER2 status, tumours expressing HIF-1α were twice as likely to be associated with nodal disease (*P *= 0.02, OR = 1.91, 95% CI = 1.09–3.33) (Table 5). HIF-1α-expressing tumours were also associated with a significantly shorter disease-free survival (*P *= 0.04, HR = 1.60, 95% CI = 1.02–2.42) (Figure 2a).

**Table 5:** Correlation analysis of hypoxia-inducible factor 1α (HIF-1α) expression and standard clinicopathological parameters

	HIF-1α-positive (*n *= 125)	HIF-1α-negative (*n *= 91)	*P *value
Median patient age (years)	59	59	0.59
Median tumour size (mm)	20	20	0.23
Tumour grade			0.52
1	22	15	
2	44	22	
3	27	23	
Nodal status			0.02
Negative	62	31	
Positive	63	60	
Oestrogen receptor status			0.90
Negative	98	72	
Positive	27	19	
Human epidermal growth factor receptor 2 status			0.77
Negative	12	11	
Positive	87	70	

*n *< 216 data not available.

### Correlation of factor-inhibiting hypoxia-inducible factor 1 with survival

Tumours with cytoplasmic FIH-1 expression were associated with a significantly worse prognosis than FIH-1 cytoplasmic-negative tumours (*P *= 0.04, HR = 1.57, 95% CI = 1.01–2.26), with a 1.8-fold increase in the risk of recurrence (*P *= 0.03, OR = 1.8, 95% CI = 1.04–2.99) (Figure 2b and Table 3). Tumours demonstrating nuclear FIH-1 expression showed a longer survival than those without nuclear expression (Figure 2c) but this did not reach statistical significance (*P *= 0.24, 95% CI = 0.53–1.17). Since this observation suggested that cytoplasmic translocation of FIH-1 from the nucleus abrogated the antagonistic effect of FIH-1, we examined the relationship among potentially functional (conserving nuclear expression) and nonfunctional (no nuclear expression) FIH-1 and survival. When stratifying tumours as nonfunctional (exclusive cytoplasmic and no FIH-1 expression) and as functional (retained nuclear expression), the group of patients with nonfunctional FIH-1 had a significantly shorter survival than those who retained FIH-1 in the nucleus (*P *= 0.02, HR = 0.42, 95% CI = 0.27–0.89) (Figure 2d).

Patients with tumours expressing FIH-1 exclusively in the cytoplasm had a significantly shorter disease-free survival compared with those expressing FIH-1 exclusively in the nucleus (*P *= 0.007, HR = 0.37, 95% CI = 0.21–0.78) (Figure 2e). Patients with tumours exclusively expressing FIH-1 within the nucleus had a significantly reduced risk of recurrence (*P *= 0.04, OR = 2.27, 95% CI = 0.20–0.96). Although not significant, patients with tumours that expressed FIH-1 exclusively in the cytoplasm demonstrated a shorter relapse-free survival than those tumours negative for FIH-1 (*P *= 0.12, 95% CI = 0.88–2.88) (Figure 2f).

When adding FIH-1 expression to standard clinicopathological factors of tumour grade, size and nodal status in multivariate analysis, neither nuclear nor cytoplasmic FIH-1 expression showed significant prognostic predictive value to the model (*P *= 0.11 and *P *= 0.14, respectively). Using the same base model, however, exclusive cytoplasmic FIH-1 expression was found to be an independent poor prognostic factor for disease-free survival, with a HR of 1.73 (*P *= 0.04, 95% CI = 1.04–2.89) (Table 6). Conversely, exclusive nuclear FIH-1 expression was associated with a longer disease-free survival (HR = 0.43, 95% CI = 0.17–1.08), although this was of borderline significance (*P *= 0.07) (Table 7).

**Table 6:** Multivariate analysis Cox regression model of disease-free survival for standard clinicopathological parameters and exclusive cytoplasmic factor-inhibiting hypoxia-inducible factor expression (*n *= 207)

	Hazard ratio	95% confidence interval	*P *value
Exclusive cytoplasmic factor-inhibiting hypoxia-inducible factor 1	1.73	1.04–2.89	0.04
Tumour grade	1.52	1.06–2.16	0.02
Tumour size	2.94	1.79–4.83	<0.001
Nodal status	2.72	1.66–4.46	<0.001

**Table 7:** Multivariate analysis Cox regression model of disease-free survival for standard clinicopathological parameters and exclusive nuclear factor-inhibiting hypoxia-inducible factor expression (*n *= 209)

	Hazard ratio	95% confidence interval	*P *value
Exclusive nuclear factor-inhibiting hypoxia-inducible factor 1	0.43	0.17–1.08	0.07
Tumour grade	1.54	1.09–2.17	0.01
Tumour size	2.89	1.76–4.76	<0.001
Nodal status	2.70	1.65–4.42	<0.001

## Discussion

In the present study we have demonstrated frequent expression of FIH-1 in the neoplastic cells of invasive breast carcinomas, with only approximately 20% of tumours being FIH-1-negative. FIH-1 was also expressed in the non-neoplastic tumour elements, suggesting a possible role in regulating HIF-1α activity in these cell populations. To the best of our knowledge there is limited data on the factors that regulate FIH-1, but it would be of interest to assess whether protein kinase Cζ, which has been shown to inhibit FIH-1 mRNA synthesis in renal cell carcinomas [32], also regulates FIH-1 expression in breast cancer.

In accordance with other studies, we observed expression of FIH-1 within the cytoplasm and nucleus of tumour cells [23,33,34]. This neoplastic cell localization was significantly associated with the phenotype of the tumour – with nuclear and cytoplasmic location being positively correlated with low and high tumour grade, respectively, and cytoplasmic FIH-1 also being associated with CA9 expression. This association suggests that nuclear FIH-1 accounts for the C-terminal TAD repression and thus leads to a reduction in HIF-1α activity. The molecular weight of FIH-1 (40.3 kDa) enables it to shuttle between cellular compartments by passive diffusion [35]. The corollary of this thesis is that FIH-1 is actively excluded from the nucleus by tumours with an aggressive phenotype. Furthermore, since there is frequent loss of chromosome 10q24 in breast cancer where the *FIH-1 *gene resides [36,37], loss of FIH-1 protein expression may occur through further mutation or epigenetic alterations in the remaining *FIH-1 *gene, as has been reported in high-grade gliomas [38]. Mutation of other HIF-1α-modifying enzymes, including prolyl hydroxylase 2 [39], have also been reported in endometrial tumours.

The regulation of HIF-1α has profound influence on the clinical outcome. High HIF-1α levels are correlated with high-grade tumours [40] and are associated with early widespread metastatic disease and survival [9]. In the present study, HIF-1α expression was positively correlated with nodal disease and a shorter disease-free survival. Although FIH-1 may indirectly affect HIF-1α levels through modulation of the PHD2 and PHD3 genes [33], FIH-1 primarily modulates HIF-1α activity rather than its level of expression [34]. This explains our observation that nuclear FIH-1 expression is positively correlated with HIF-1α expression, since FIH-1 is not involved in the degradation of HIF-1α. Interestingly, FIH-1 appeared to be associated with HIF-1α regardless of its subcellular location, although the significance of its association with FIH-1 in the cytoplasm is not clear.

As FIH-1 is able to modulate HIF-1α activity, it would be expected to affect tumour behaviour in a similar manner. Our finding that nuclear and cytoplasmic FIH-1 expression is associated with a reduced risk and an increased risk of recurrence, respectively, is in keeping with hydroxylation of HIF-1α in the nuclear compartment, thereby interfering with coactivator binding through the CH-1 domain of p300. We have used subset analysis to assess the potential effects of FIH-1 on outcome because of the clear differences in expression patterns that are likely to be functionally important. Indeed, we have previously shown that breast cancer using this compartmentalization process to enhance coactivator recruitment for cytoplasmic localization of CBP/p300-interacting transactivator (CITED) -4, a new member of the CITED family that can compete with HIF-1α for p300, has a worse prognosis [41]. In an analogous manner, inhibiting FIH-1 through translocation to the cytoplasmic compartment would allow an enhanced hypoxic response mediated through HIF-1α.

The above findings suggest that modulation of FIH-1 would be a powerful mechanism to reduce the HIF response. This is a particularly attractive target since, unlike the prolyl hydroxylases, FIH-1 can reduce HIF-1α activity over a range of oxygen tensions and even in severely hypoxic conditions [21,33]. Agents targeting FIH-1 would therefore further complement the repertoire of therapeutic agents aimed at inhibiting HIF-1α activity in tumours. Proof of principle for the potential of such intervention is shown by amphotericin B, a commonly used treatment for systemic mycoses, which enhances the interaction between HIF-1α C-terminal TAD and FIH-1, thereby blocking p300 recruitment and suppressing hypoxia-induced erythropoietin production [42]. It is likely that the repressive signals mediated by FIH-1 go beyond that of changing the HIF response since FIH-1 has recently been shown to hydroxylate the IκB family at specific asparaginyl residues that are conserved but present in a range of ankyrin repeat domains [43]. These data demonstrate the potential of FIH-1 to modulate the clinical outcome in tumours.

## Conclusion

In the present study we have demonstrated a widespread expression of FIH-1 among invasive breast carcinomas, with particular subcellular locations of FIH-1 being associated with prognosis. Our findings suggest the value of further studies to investigate the mechanism of nuclear localization of FIH-1 and how it can be manipulated to target the HIF-1α pathway and other pathways in breast cancer therapy.

## Abbreviations

CA9 = carbonic anhydrase 9; CI = confidence interval; ER = oestrogen receptor; FIH-1 = factor-inhibiting hypoxia-inducible factor 1; H & E = haematoxylin and eosin; HER2 = human epidermal growth factor receptor 2; HIF = hypoxia-inducible factor; HR = hazard ratio; OR = odds ratio; PBS = phosphate-buffered saline; PHD = prolyl hydroxylase domain; TAD = transcriptional activation domain.

## Competing interests

The authors declare that they have no competing interests.

## Authors' contributions

YET performed data analysis and manuscript preparation; LC and HT were responsible for tissue microarray construction and immunohistochemistry; CH performed statistical analysis; FP and KCG were responsible for the scoring of stained tissue arrays ALH provided useful advice and comments and SBF was responsible for concept, design, data analysis and manuscript preparation.
